# Evaluating the Efficacy of Peroneus Longus Tendon as an Autograft for Anterior Cruciate Ligament Reconstruction in Indian Female Patients: A Prospective Cohort Study

**DOI:** 10.7759/cureus.71665

**Published:** 2024-10-16

**Authors:** Anoop Pilar, Sandesh GM, Mevin M Nedumparampil, Harish Kodi, Rajkumar Amravathi, Madan Mohan Muniswamy

**Affiliations:** 1 Orthopaedics, St. John's Medical College Hospital, Bengaluru, IND; 2 Orthopaedic Surgery, St. John's Medical College Hospital, Bengaluru, IND; 3 Orthopaedics and Traumatology, St. John's Medical College Hospital, Bengaluru, IND

**Keywords:** acl tear, females, functional outcome, non-athlete, peroneus longus

## Abstract

Background

Anterior cruciate ligament (ACL) injuries are prevalent, especially among active individuals, and are increasingly observed even in Indian women who are not active in sports. ACL reconstruction (ACLR) is commonly performed using various graft options, but optimal choices for women remain debated. The aim of our study was to examine the use of the peroneus longus tendon as an autograft for ACLR in Indian female patients.

Materials and methods

A prospective cohort study was carried out on 44 non-athlete female patients who met the inclusion criteria. The study evaluates functional outcomes using the International Knee Documentation Committee (IKDC), Lysholm-Tegner, and American Orthopaedic Foot and Ankle Society (AOFAS) scores, as well as knee stability through clinical tests, over a one-year postoperative period.

Results

The study demonstrates significant improvements in knee function, with substantial increases in IKDC and Lysholm-Tegner scores from preoperative levels (p-value < 0.01), and effective restoration of knee stability, as shown by improved Lachman, Anterior Drawer, and Pivot Shift test results. Moreover, the peroneus longus graft had minimal impact on ankle morbidity, as indicated by consistent AOFAS scores before and after surgery.

Conclusion

The study highlights the peroneus longus tendon as a viable graft option for ACLR in symptomatic Indian female patients who were not active in sports, offering both mechanical strength and favorable functional outcomes without affecting the ankle strength or the function.

## Introduction

Anterior cruciate ligament (ACL) injury is common,among active individuals. ACL tears contribute to approximately 50% of knee injuries [1]. With the number of injuries increasing, the number of ACL reconstruction (ACLR) procedures is also on the rise, especially in children, adolescents, and women. The female gender has a two- to eight-fold increase in the incidence of ACL injuries compared with the male [2]. The causes can be intrinsic or extrinsic. Extrinsic causes include the biomechanics of body movements, muscle strength, shoe surface interface, and skill levels. Intrinsic causes include joint laxity, hormonal, limb alignment, and increased posterior tibial slope causing mechanical valgus loading with internal rotation of the tibia, notch dimensions, and ligament size. The precise roles that hormonal and genetic variables may play in female ACL injuries are unknown but the hormonal fluctuation during the menstrual cycle increases ligament laxity and decreases neuromuscular performance.

The Indian woman measures an average of 150-160 cm in height [3]. The length and diameter of the soft tissue hamstring graft available for ACLR are not adequate. No single graft choice has been shown to have superior outcomes in women. The topic remains an area of debate [4]. Even though bone-patellar tendon-bone (bone-tendon-bone (BTB)) grafts provide advantages such as bony fixation and incorporation, strong structural properties, and predictable success rate in restoring knee stability, it is often associated with the risk of anterior kneeling pain, patellar fracture, with an invasive approach and a large incision causing cosmetic issues in women, fixed length and a weaker than native ACL. There is disagreement over the best surgical therapy, including graft selection. Every graft option that is currently available has advantages and disadvantages [5]. No high-level studies have documented a superior graft choice in Indian women.

The peroneus longus tendon (PLT) has garnered interest as a graft option for ACLR due to its suitable size and mechanical properties. It can be used as the autograft as the peroneus brevis and peroneus longus have a synergistic action; hence the donor site morbidity is reduced [6]. Harvesting the PLT is a relatively straightforward procedure, with limited donor site morbidity. Compared to traditional autografts like the patellar or hamstring tendon, the peroneus longus graft may offer improved proprioception and an earlier return to activity [7].

Non-sporting women are a unique patient population that warrants further investigation, as they may have different functional needs and activity levels compared to those who are active in sports. Although the peroneus longus graft has shown promising results in select patient populations, the functional outcomes in Indian female non-athlete patients are not well-established. Thus, this study aims to examine the functional outcomes of ACL reconstruction using the peroneus longus graft in an Indian female population that has symptoms of instability, plays recreational sports, does household activities, and works in the office, which requires climbing stairs, brisk walking, and running.

## Materials and methods

This was a prospective cohort study conducted from November 2020 to November 2022 at St. Johns Medical College Hospital, Bengaluru, India. The study was approved by the Institutional Ethics Committee of St. Johns Medical College Hospital (approval number: IEC/1/188/2022), and informed consent was secured from all participants. All non-athlete, female patients who fulfilled the inclusion criteria during the study period were selected. We included a total of 44 patients in the study.

Inclusion and exclusion criteria

All female patients with ACL tears who were symptomatic and played recreational sports or did household activities or office work that required climbing stairs, brisk walking, and running, were considered for the study. Inclusion criteria were strict, targeting individuals with isolated ACL tears, demonstrating Grade 2 and 3 laxities on the Anterior Drawer and Lachman tests and MRI-confirmed complete ACL tears. Participants selected for the study were those with no previous injuries or surgeries to the ipsilateral ankle, knee, or hip joint, and who presented with normal foot arches. Only patients with acute ACL injuries who followed a preoperative rehabilitation protocol for three weeks were included. This protocol involved wearing knee immobilizers and engaging in physical therapy designed to restore knee function. Specifically, these exercises aimed to achieve a nearly full range of motion, symmetric quadriceps strength, and reduced joint effusion. The preoperative physical therapy regimen also included exercises to activate the peroneal muscles, ensuring the knee was stable and the inflammatory phase had passed before surgery was scheduled.

Exclusion criteria were stringent, disqualifying patients with partial thickness ACL tears, multi-ligament injuries, concurrent meniscus injuries, and those requiring revision ACL reconstruction. Professional athletes and individuals with paralytic conditions like poliomyelitis or learning disabilities were also excluded from the study. Before surgery, all patients underwent a series of assessments, including Tegner-Lysholm [8], International Knee Documentation Committee (IKDC) [9], and American Orthopaedic Foot & Ankle Society (AOFAS) [10] scores to establish baseline functional status.

Operative technique

All surgeries were performed under spinal anesthesia. The surgical procedure began with a diagnostic arthroscopy to confirm the complete tear of the ACL. The PLT was chosen as the autograft, and a harvesting technique was employed that involved a 1-2 cm incision on the posterior aspect of the lateral malleolus. Through this incision, the peroneal sheath was identified, isolated, and incised. The entire PLT was then harvested using a tendon stripper and subsequently tripled to increase its diameter and strength. Notably, no tenodesis was performed to secure the remaining peroneus longus to the peroneus brevis, allowing for a quicker and less invasive recovery at the donor site. The peroneal sheath was closed with absorbable sutures, and the incision was closed with non-absorbable sutures.

Once the autograft was prepared and sized, the standard anatomical femoral tunnel was drilled using the inside-out technique via the accessory anteromedial portal, while the tibial tunnel was drilled using the outside-in technique. For fixation, all patients received femoral fixation with an adjustable suspensory fixation device, and tibial fixation was achieved using a biodegradable screw. Following fixation, the graft's stability was assessed both arthroscopically by probing the ligament and clinically through the Lachman’s test and the anterior drawer test. In pathologies in the knee like meniscus tear, cartilage injuries were addressed in the standard techniques. The surgical incisions and portals were then closed with non-absorbable sutures.

Postoperative care and rehabilitation

Immediately following surgery, all patients were placed on a structured, short-term physiotherapy regimen divided into six phases as per the standard Melbourne ACL Rehabilitation Guide 2.0, which was used from day one postoperatively. The rehabilitation program included a variety of exercises aimed at restoring knee function and minimizing complications as follows: (i) Active Assisted to Active Motion: Exercises were initiated as tolerated by the patient to promote early movement; (ii) Passive Extension Exercises: Techniques such as heel press, leg hanging in the prone position, and active-assisted extension were employed to restore full extension; (iii) Passive Flexion Exercises: Gradual flexion exercises were introduced to maintain and improve range of motion; (iv) Quadriceps Strengthening Exercises: These included isometric quadriceps contractions, straight leg raises (SLR), controlled terminal knee extensions, and both concentric and eccentric contractions to strengthen the knee; (v) Ankle and Foot Exercises: Dorsiflexion, plantarflexion, active toe movements, as well as inversion and eversion movements were incorporated to ensure ankle mobility and prevent stiffness; (vi) Full Weight-Bearing Walker Assistance: Patients were encouraged to begin walking with the assistance of a walker while bearing full weight on the affected leg to promote normal gait patterns.

Patients returned for follow-up on postoperative day 12 for suture line inspection and removal of staples or sutures. During this visit, the assessment parameters included evaluating the suture line for healing, checking for any signs of swelling or effusion, assessing the surrounding skin condition, and evaluating the range of motion in both the knee and ankle. The use of a knee immobilizer was continued until the patient demonstrated good quadriceps control, particularly in performing active SLRs and dynamic quadriceps exercises without any extensor lag. This level of control was typically achieved by approximately four weeks postoperatively.

Follow-up and assessment

Patients were closely monitored at regular intervals following surgery. Functional assessment of the knee was conducted in a blinded manner by a single examiner, using the Tegner-Lysholm knee score at six months and one year postoperatively. Ankle morbidity was evaluated using the AOFAS score, and knee stability was clinically assessed using the Anterior Drawer, Lachman, and pivot shift tests at both the six-month and twelve-month follow-up appointments.

Statistical analysis

All collected data were compiled and analyzed using IBM SPSS Statistics for Windows, Version 24.0 (Released 2016; IBM Corp., Armonk, New York, United States), allowing for a comprehensive evaluation of the improvements observed between the two follow-up periods, the overall functional outcomes of the reconstructed knee, and the condition of the donor ankle. Age, BMI, and level of physical activity (categorized as recreational vs. non-athletic) were recorded for each patient.

## Results

The study cohort consisted predominantly of patients over 30 years of age, accounting for 54.5% of the sample, with the mean age being 27.67 ± 7.98 years. The majority of the injuries were domestic (82%), and a larger proportion of the injuries affected the right knee (59%). The mean graft diameter used was 8.75 ± 0.24 mm, reflecting the suitability of the PLT as an adequate graft source (Table 1).

**Table 1:** Patient demographics, sports participation, injury characteristics

Characteristics		Count	Percentage
Age	<20 years	4	9.1%
	21-30 years	16	36.4%
	>30 years	24	54.5%
	Mean ± SD (years)	27.67 ± 7.98	
Mechanism	Domestic	36	82%
	Recreational	8	18%
Side	Left	18	41.0%
	Right	26	59.0%

The functional outcomes showed significant improvement postoperatively, as evidenced by the IKDC and Lysholm-Tegner scores. Preoperative IKDC scores averaged 57.05 ± 5.87, which improved significantly to 85.00 ± 12.65 at six months postoperatively and further to 91.40 ± 11.59 at 12 months (p < 0.001 for both comparisons). Similarly, the Lysholm-Tegner score improved from 73.67 ± 8.76 preoperatively to 90.87 ± 9.84 at six months and 95.47 ± 6.91 at 12 months (p < 0.001 for both comparisons).

The AOFAS scores, which were used to assess ankle morbidity due to PLT harvesting, did not show significant differences between preoperative and postoperative values. The mean AOFAS score was 93.16 ± 2.67 preoperatively, 92.43 ± 3.65 at six months, and 92.87 ± 2.78 at 12 months, with p-values of 0.084 and 0.082, respectively (Table 2).

**Table 2:** Comparison of preoperative and postoperative functional scores IKDC: International Knee Documentation Committee; AOFAS: American Orthopaedic Foot & Ankle Society *p-value is significant

Functional Score	Mean	SD	P value
IKDC			
Preoperative	57.05	5.87	<0.001*
Postoperative, 6 months	85.00	12.65	<0.001*
Postoperative, 12 months	91.40	11.59	<0.001*
Lysholm -Tegner			
Preoperative	73.67	8.76	<0.001*
Postoperative, 6 months	90.87	9.84	<0.001*
Postoperative, 12 months	95.47	6.91	<0.001*
AOFAS			
Preoperative	93.16	2.67	<0.001*
Postoperative, 6 months	92.43	3.65	<0.084
Postoperative, 12 months	92.87	2.78	<0.082

Knee stability assessments revealed a marked improvement postoperatively. Preoperatively, 70.5% of patients had a Grade 3 Lachman test, indicating severe knee instability. This number decreased to 6.9% with a Grade 1 Lachman test at six months postoperatively, and by 12 months, 95.5% of the patients exhibited Grade 0 Lachman test results, indicating normal knee stability.

The Anterior Drawer test results mirrored these findings. Preoperatively, 88.6% of patients demonstrated Grade 3 instability, which improved to 4.5% with Grade 1 instability at six months, and by 12 months, all patients (100%) had Grade 0 results. Similarly, the Pivot Shift test, which is a crucial indicator of rotational stability, showed significant postoperative improvements. Preoperatively, 95.5% of patients had Grade 2 results, but at both six and 12 months postoperatively, all patients (100%) exhibited Grade 0 results, indicating no rotational instability.

## Discussion

The study provides valuable insights into the use of the PLT as an autograft for ACLR in a female population in India. The findings support the PLT as a viable graft option, demonstrating significant improvements in knee stability and function postoperatively while maintaining minimal donor site morbidity.

Historically, ACLR has predominantly utilized autografts like the BTB and hamstring tendons. A prior biomechanical investigation revealed no apparent difference in tensile strength between the four-strand hamstring and peroneus longus grafts. The diameter of the graft is one of the most crucial factors during knee ACLR surgery. When quadrupled-strand hamstring autografts with a minimum diameter of 8 mm are used for ACLR, failure rates are significantly minimized. A patient who is shorter than 149 cm in height needs graft augmentation since their prospective graft size is less than 7 mm [11].

According to the Annals of Joint 2021 recommendations, to optimize ACLR in the female athlete it is recommended to use a graft size of >9 mm robust graft or one-third of the diameter of the lateral wall of the lateral femoral condyle [12].

A study by Mariscalco et al. found a substantial positive correlation between higher Knee Injury and Osteoarthritis Outcome Score (KOOS) and IKDC scores, as well as higher revision rates when the graft size was less than 8 mm, and a 1 mm increase in graft diameter. However, the search for an optimal graft choice, particularly in female patients, remains a challenge [12]. The PLT is a promising alternative for ACLR, offering sufficient tensile strength, consistent graft diameter, and minimal donor site morbidity. In our study, the average graft diameter exceeded 8 mm, indicating its potential to provide adequate knee stability and reduce the risk of re-rupture. This makes it particularly suitable for non-athlete women who require a stable knee for daily activities without the high demands of athletic performance [13].

The study's findings highlight how effectively the PLT restores knee function after ACLR. The capacity of the graft to regain knee stability and function to pre-injury levels is demonstrated by the significant increase in the IKDC and Lysholm-Tegner scores after surgery (Table 2). Prior research has demonstrated advantages for both functional outcome and knee stability following ACLR using the PLT [14]. The results obtained demonstrate that the PLT can be used in single-bundle ACLR with good-to-exceptional functional outcomes at the patient's one-year follow-up. Even while it was believed that a longer short-term evaluation (at least two years) would be required, a previous study showed that there were barely any differences in functional outcomes between the one- and two-year follow-ups following ACLR [15]. The PLT is recommended since patients do not experience patellofemoral discomfort or loss of extension or flexion. This is especially remarkable considering the patient population, which was predominately older, non-athletic, and of the female gender. These functional scores significantly improved from preoperative levels to six and 12 months postoperatively, indicating that the graft can support early recovery.

Furthermore, there were no significant differences between preoperative and postoperative values in the AOFAS scores, which were utilized to evaluate ankle morbidity as a result of PLT harvesting. We discovered that the functional outcome was still outstanding despite donor site morbidities such as lower peak torque eversion and inversion, poorer ankle function, and concerns regarding ankle stability, similar to the findings in the PL group in a study by Angthong et al. [16]. Ankle instability or decreased movement was not seen by any patients in a study conducted by Vijay et al. and the mean ankle eversion strength at the donor site did not differ significantly from the contralateral normal ankle [17]. Similar to this, Rhatomy et al. reported that after one year, the AOFAS score was high, demonstrating that ankle functions were substantially unaffected by PLT harvesting [18]. None of our patients complained of something like this. This is explained by the peroneus brevis's superiority in ankle eversion as well as the harvested graft's capacity for regeneration [19]. It indicates that the morbidity of the ankle joint is mostly unaffected by either the excision or the harvesting of the PLT.

The improvements in knee stability postoperatively, as indicated by the Lachman, Anterior Drawer, and Pivot Shift tests, are also significant. Preoperatively, a majority of patients exhibited severe knee instability, with a high percentage showing Grade 3 results in these tests. Postoperatively, there was a marked reduction in instability, with most patients achieving Grade 0 results by the 12-month follow-up. Our study produced similar results to those of Agarwal et al., who also reported similar results with the Lachman and Anterior Drawer tests [20]. These findings indicate that the PLT provides sufficient strength and stability to the reconstructed ACL, comparable to or even surpassing more traditional graft options.

Limitations of the study

While the study shows promising results with the PLT for ACLR, it has several limitations. The one-year follow-up period does not fully capture long-term outcomes, such as potential re-rupture or graft failure, and longer studies are needed to confirm the durability of these results. The focus on non-athlete female patients limits the generalizability of the findings to other populations, like athletes or males. Additionally, the study did not assess long-term effects on ankle stability and mobility or directly compare the PLT with other graft options, leaving some uncertainty about its relative efficacy and safety.

Future studies should also explore the biomechanical properties of the PLT in more detail, comparing it directly with other graft options in larger, randomized controlled trials. Such studies would help to establish a more definitive understanding of the PLT’s efficacy and safety, particularly in comparison to traditional graft choices.

## Conclusions

The study successfully demonstrates the potential of the PLT as a reliable and effective autograft for ACLR in the Indian female population that has symptoms of instability, plays recreational sports, does household activities, and works in an office that requires climbing stairs, brisk walking, and running. The graft offers significant improvements in knee function and stability, with minimal impact on the donor site. However, further research is needed to fully establish its long-term efficacy and safety across different populations. The findings of this study contribute to the ongoing debate over the optimal graft choice for ACLR, particularly in women, and suggest that the PLT may be a viable alternative to traditional graft options.
